# HIV Infection in the Elderly: Arising Challenges

**DOI:** 10.1155/2016/2404857

**Published:** 2016-08-09

**Authors:** Bonaventura C. T. Mpondo

**Affiliations:** Department of Internal Medicine, School of Medicine, College of Health Sciences, The University of Dodoma, Dodoma, Tanzania

## Abstract

Globally there is an increase in the number of people living with HIV at an advanced age (50 years and above). This is mainly due to prolonged survival following the use of highly active antiretroviral therapy. Living with HIV at an advanced age has been shown to be associated with a number of challenges, both clinical and immunological. This minireview aims at discussing the challenges encountered by elderly HIV-infected patients.

## 1. Introduction

Globally the number of elderly HIV-infected patients is increasing [1]. Estimates show that the percentage of people living with HIV aged 50 years and above had increased to more than 17% over the past decade [2]. In sub-Saharan Africa (SSA), estimates show that the percentage will triple by the year 2040 [3]. Previous data showed that HIV associated morbidity and mortality was higher in elderly patients as compared to their younger counterparts [4, 5]. More recent studies have also found that old age is associated with rapid progression to AIDS [6, 7]. Treatment response may also differ between elderly patients and younger patients. Several reports have demonstrated impaired immune recovery in elderly patients [8, 9]. Toxicities to antiretroviral drugs are more likely in elderly patients than in younger ones. Elderly patients are also more likely to have comorbidities including cardiovascular disease, renal disease, and diabetes.

## 2. Epidemiology

Since its discovery in 1980s, HIV has largely been considered as the disease of young people. However, recent reports indicate that the epidemiology of the infected population is fast changing, with significant increase in the number of elderly patients [1]. The number of people living with HIV has been shown to be on the rise in both developed countries as well as developing countries [3, 10]. Projections indicate that the number will continue to rise in the coming decades [3]. In 2007, it was estimated that approximately 15% of all HIV-infected individuals were aged 50 years and above in Tanzania [11]. The proportion remained stable in another report in 2014 [12]. In HIV/AIDS, the age of 50 years and above is considered to be old age because it was much older when compared to the lower mean age of HIV-infected patients early in the epidemic [13].

One of the reasons for the epidemiological change in the population living with HIV is the longevity of life following the use of highly active antiretroviral therapy (HAART). With the use of HAART, HIV has become a chronic manageable disease [14]. The causes of morbidity and mortality are no longer AIDS related, but rather complications from cancers, renal diseases, liver diseases, and the like [15]. There are also reports that show increased risk of acquiring HIV in elderly individuals [16, 17].

When compared to younger patients, elderly patients are usually diagnosed at an advanced stage of HIV [18, 19]. Several factors have been associated with late or missed diagnosis in this population. These factors include lack of awareness of HIV risk factors in this population, failure of health care providers to suspect HIV in this population, absence of routine HIV screening in this population, and overriding of HIV symptoms with symptoms of other common conditions associated with aging [16, 20].

## 3. Effects of Aging and HIV Infection on the Immune System

### 3.1. Effects on T Cell Function

As a person gets old (45 years and above), involution of the thymus occurs which results to a decrease in thymic volume [21]. The thymus is an organ that is involved in the development of human immune system and takes part in T lymphocyte maturation. Apart from the contraction of thymic volume, the production of naïve T cells decreases with increasing age [22]. There is also an association between increasing age reduced T cell functionality, decreased population of memory T cells, and reduced number of functional cytotoxic T cells [23].

HIV may also directly affect the immune system. HIV infection may inhibit the function of thymus and the production of naïve T cells [24]. Therefore HIV compounds the effect of aging on the immune system. This explains why the progression of HIV in elderly patients is usually more pronounced. In one case control study, it was found that both HIV-infected and noninfected elderly individuals had significantly lower proportions of functional cytotoxic T cells when compared to younger patients; the lowest proportions being in HIV-infected elderly patients [21]. Since cytotoxic T cells are involved in inhibiting HIV replication, reduction in the number and functionality of these cells coupled with the loss of CD4 cells leads to accelerated progression of HIV in elderly patients. Several studies have found that age is a predictor of HIV progression [25].

### 3.2. Effects on B Cells and Antibody Function

HIV infection and aging affect not only T cell function, but also the B cell functions [26]. There is evidence of impaired B cell functioning in both HIV-infected and older adults. With old age or HIV infection, evidence shows that there is a decrease in naïve B cell reserve. There is also evidence of depletion of memory B cells from peripheral blood in HIV-infected individuals [27]. In HIV-infected patients, studies have shown that there is abnormal B cell activation and immunoregulation [26, 28]. The effect of age on B cell activation remains unclear [29]. In both HIV-infected patients and older adults, the ability of antigens to activate B cells and generate effective antibody response is impaired [30].

## 4. Response to HAART

Data regarding the clinical, immunological, and virological benefits of HAART in elderly HIV-infected patients have been conflicting. Early in the HAART era, data suggested that age was inversely proportional to the rate of immune system recovery [9, 31]. This means that the immune recovery in older patients was lower than that in younger patients. This was hypothesized to be related to the reduction in thymic size and function [32–34].

Studies comparing immunological and virological responses among elderly and younger patients have come up with mixed results. Some studies concluded that CD4 response in elderly patients was lower compared to younger patients [8, 9, 35]; however other studies did not find any significant difference [36, 37]. A study done in Tanzania found a significant difference in absolute CD4 gain between elderly and younger patients with a lower gain among elderly patients [38]. Controversy also remains regarding virological response when the two populations are compared. Some studies indicated better response in elderly patients [37, 39]; another study showed better response in younger patients [40]; some studies did not find any significant difference between the two populations [31, 41]. Results from the largest cohort study comparing virological and immunological response found that virological response was better in elderly patients; however the immunological response was poor in this population [42]. The study also found poor clinical outcomes in elderly patients as compared to younger patients. Several studies have concluded that mortality rates are higher in elderly patients than in young patients [42, 43]. The causes of death were noted to be non-HIV-related complications. Studies evaluating immunological and clinical responses to HAART are summarized in Table 1.

## 5. Functional and Metabolic Complications of Aging with HIV Infection

### 5.1. HAART Metabolism and Toxicity

Data on the toxicities of antiretrovirals in elderly patients is very limited. This is due to the fact that most studies on antiretroviral metabolism exclude patients with advanced age with comorbid conditions. The pathophysiology of HAART toxicity is believed to be multifactorial; however, metabolic abnormalities and metabolic and endocrinological disorders are believed to be the main mechanisms.

Physiologically, there is a decline in creatinine clearance with increasing age. This reduction can potentially affect the metabolism of renally excreted drugs. There is also a challenge in estimating renal function in elderly patients. Both old age and HIV can lead to reduction in muscle mass making estimation of renal function using serum creatinine very difficult and unreliable [47]. Drugs such as nucleoside reverse transcriptase inhibitors (NRTIs) which are the backbone of HIV treatment regimen in Africa are eliminated by the kidneys through both tubular secretion and glomerular filtration; these need dose adjustments in patients with renal insufficiency.

Drug pharmacokinetics can also be influenced by the age related changes in body composition. The decrease in body weight and in total body water leads to change in drug volume of distribution which in turn leads to increased concentration of drugs in blood and other tissues [48]. There is also a possibility of delayed onset of drug effects due to slower gastrointestinal absorption rate in elderly patients [49]. Protein bound drugs usually have enhanced effects in elderly patients who have decline in protein concentrations as they age.

Tenofovir, which constitutes the first-line regime in most African countries, is nephrotoxic and may exacerbate renal insufficiency in elderly patients especially when used in combination with protease inhibitors (PIs) [50]. Most of the PIs and nonnucleoside reverse transcriptase inhibitors (NNRTIs) can potentially exacerbate hepatic insufficiency especially in patients with preexisting liver disease [51].

### 5.2. Comorbid Disease States

With the use of HAART, HIV has become a chronic and manageable condition. Rates of opportunistic infections (OIs) have declined significantly; however the non-HIV-related complications have been noted to be on the rise especially in elderly patients [14]. Conditions such as diabetes, cerebrovascular diseases, ischemic heart diseases, liver diseases, renal diseases, and cognitive abnormalities have been on the rise in elderly HIV-infected patients. Compared with HIV-negative elderly patients, HIV-infected elderly patients show accelerated aging [52, 53]. Age related complications are more common and present earlier in HIV-infected than in HIV-negative elderly patients as a result [54].

#### 5.2.1. Metabolic Changes and the Risk of Cardiovascular Disorders

HIV infection and some of the antiretroviral drugs have been associated with fat and metabolic changes which increase the risk of cardiovascular disorders (CVDs) [55, 56]. Both nucleoside reverse transcriptase inhibitors, specifically stavudine that has been phased out and the protease inhibitors, have been associated with lipodystrophy [57, 58]. Some studies found association between lipodystrophy and age [58]. There is also evidence of increasing prevalence of CVDs with increasing age among HIV-infected individuals [59].

Evidence suggests that age is an independent predictor for cardiovascular diseases. Studies have shown that there is accumulation of atherosclerosis which predicts the chances of future development of cardiovascular disease [60]. The role of HAART in causing cardiovascular disorders remains unclear. While other studies have concluded that there is an association between the use of HAART and cardiovascular events [61, 62], others have not found an association [63, 64]. In one study, rates of hypertension were higher in ART naïve HIV-infected patients 1 year after starting HAART [65].

#### 5.2.2. Kidney and Liver Diseases

With aging, there is physiological decline in renal and liver functions. Hepatic and renal insufficiencies are therefore common in HIV-infected elderly patients. With the increasing prevalence of diabetes, hypertension, and hepatitis C viral infection, the prevalence of renal insufficiency will further increase in this population. Old age, hepatitis C coinfection, black race, and advanced HIV have been associated with kidney disease [66]. The prevalence of kidney diseases increases with both age of the HIV-infected individuals and the type of HAART used [67, 68]. Both old age and HIV can lead to reduction in muscle mass making estimation of renal function using serum creatinine very difficult and unreliable [47].

HIV-infected elderly patients are predisposed to hepatic insufficiency. With aging, the size of the liver decreases; the blood flow to the liver decreases as well [69]. Coinfection between HIV and viral hepatitis is common. Studies show that between 10% and 20% of HIV-infected individuals are coinfected with hepatitis B [70, 71]. One global meta-analysis found consistently higher hepatitis C virus prevalence in HIV-infected individuals than* *HIV-negative individuals* *across all risk groups and regions [72].

Even in the HAART era, morbidity and mortality associated with liver disease among HIV-infected people remain high [73]. Liver related morbidity and mortality among HIV-infected people are four times higher in elderly patients than younger ones [74]. The risk of liver cirrhosis is higher in patients coinfected with HIV and viral hepatitis [75]. In one study both HIV infection and old age were associated with progression to end stage liver disease in patients with hepatitis C [76]. There is no evidence on the best ways to manage viral hepatitis in HIV-infected people. Hepatic insufficiency is common in HIV-infected elderly patients; screening for liver function and viral hepatitis is important in this population.

#### 5.2.3. Non-AIDS-Defining Cancers

Prior to the use of HAART, the prevalence of AIDS-defining malignancies (ADMs) (e.g., non-Hodgkin's lymphoma, Kaposi's sarcoma, and invasive cervical carcinoma) was high and was directly correlated with the degree of immunosuppression. With the rapid scale up of HAART, however, the prevalence of ADM has significantly declined [77]. In the HAART era, the incidence of non-AIDS-defining malignancies (NADMs) has been on the rise [77, 78]. These include lung, liver, brain, and anal cancers which have been found to be more common in HIV-infected than in HIV-negative individuals [79, 80]. Most of the NADMs unlike ADMs are not correlated with the degree of immunosuppression [77]. Although the rate of ADMs continues to decline in the HAART era, the rate of NADMs continues to rise and now accounts for the majority of cancers in HIV-infected persons [81]. The development of NADMs is associated with increasing age among HIV patients [81]. HAART use is therefore protective for ADMs, but does not seem to significantly impact the incidence of NADMs.

#### 5.2.4. Neuropsychiatric Manifestations

There is also an issue of neuropsychiatric manifestations of HIV [82]. HIV infection is considered the most common cause of preventable neurocognitive abnormalities [83]. It is estimated that between 30% and 50% of HIV-infected individuals will experience some form of neurocognitive decline [84] ranging from mild decline in motor and information processing decline to severe dysfunction impairing activities of daily living (ADLs) [85].

Neurocognitive abnormalities have been found to be more common in HIV-infected elderly patients than in age matched HIV-negative patients. Studies show that elderly patients are at an increased risk of developing cognitive impairment as compared to younger patients [86]. In elderly patients, depressive symptoms and low CD4 count have been found to be the risk factors for cognitive impairment [87]. Besides the higher prevalence of dementia in older versus younger adults, differences also have been found in milder cognitive diagnoses, with 44.7% of the older group and 26.3% of the younger group meeting formal criteria for minor cognitive motor disorder (MCMD).

### 5.3. Polypharmacy and Potential Drug to Drug Interactions

Studies have shown an increase in comorbid and multimorbid non-AIDS conditions such as diabetes mellitus, hypertension, osteoporosis, and cardiovascular diseases among elderly patients living with HIV [88]. Elderly HIV patients are faced with the challenge of consuming multiple drugs for the treatment of both HIV and the comorbid conditions. Combination antiretroviral therapy, which consists of at least three medications, together with medications needed to treat comorbidities makes this population at high risk for polypharmacy [89–91] which is defined as the concomitant use of multiple drugs commonly ≥5 different drugs [92, 93]. One review found that the prevalence of polypharmacy is increasing in elderly patients and is associated with increased morbidity and mortality [94].

Patients taking multiple drugs have an increased risk for falls, drug to drug interactions (DDI), adverse drug effects (ADE), increased hospitalization, and poor drug adherence [92]. Studies have shown that HIV elderly patients with multiple medication are at an increased risk of having DDI than the younger patients [91]. Because of the physiological changes that alter drug metabolism, drug toxicities are also exacerbated in these patients when they use several types of drugs concomitantly. Health care providers should therefore be aware of the risks and fully evaluate all medications at each patient visit to prevent polypharmacy from occurring in HIV-infected elderly patients who are at increased risk.

### 5.4. Frailty, Functional Impairment, and Disability

#### 5.4.1. Frailty

Frailty is defined as a state of increased vulnerability in old age due to diminished physical reserve [95, 96]. Its phenotype is defined by presence of three of the following features: exhaustion, low activity level, weakness, weight loss, or slowed walking speed [97]. Studies have shown that frailty is associated with poor health outcomes including hospitalization, morbidity, and mortality among affected individuals [98]. The exact mechanism for frailty remains unclear; however there is association with increased free radical levels, mitochondrial dysfunction, and cytokines that might activate inflammatory pathways. In older individuals with frailty phenotype, the levels of C-reactive protein, D-dimer, fibrinogen, and IL-6 have been found to be elevated [99].

Overlap between HIV and frailty has been reported since early in the AIDS epidemic. Frailty continues to be most frequently recognized among HIV-infected persons not on ART, with low CD4 T cell count, or high HIV-1 RNA [100]. The prevalence of frailty in routine HIV care has been reported to be between 5% and 33%. It was also observed in one study that frailty occurs earlier in HIV-infected than in HIV-negative individuals [97]. Old age has been found to be one of the factors associated with frailty [100–102]. A study done in South Africa found significantly higher rates of frailty among HIV-infected adults than in HIV seronegative ones; in this study old age was a significant predictor of frailty [100].

#### 5.4.2. Functional Impairment

Functional impairments in HIV/AIDS were initially reported in patients with advanced HIV. Tools used to assess functional impairment are used to identify differences in exercise tolerance, grip strength, balance, gait speed, and chair rise time in HIV-infected individuals. Previous studies assessing functional impairment in HIV-infected patients showed that middle-aged, HIV-infected men on HAART have reductions in exercise capacity, functional performance, physical activity, and grip strength [103]. HIV-infected elderly patients usually present with comorbid conditions, sometimes multimorbid, related to aging and the serostatus. One cohort study found that age-associated comorbidity affects physical function in HIV-infected patients and may modify the effect of aging [104].

#### 5.4.3. Disability

Increased risk of disability among HIV-infected patients was described early in the epidemic. Disability describes the difficulty or dependency in carrying out activities in the environment. Reports on disability exist both from pre-ART and in the era of effective ART. One of the largest studies assessing disability in pre-ART era found that 14% of study participants reported difficulties in independent activities of daily living (IADLs) only and 4% in both activities of daily living (ADLs) and IADLs [105]. In the era of ART, several studies have also found higher rates of disability in HIV-infected patients [106, 107]. In these studies, age and HIV status had synergistic effects in causing disability [108].

## 6. Aging with HIV in Africa

With the rapid scale up of ART, the number of HIV-related deaths has declined significantly in SSA [109]. As a result, the number of people living with HIV at an advanced age has been projected to increase significantly in the coming years [110]. Despite the increasing number of elderly HIV-infected patients, very little data has been published over the subject. Studies addressing the challenges of living with HIV at an advanced age in Africa are scarce [111].

One of the challenges of aging with HIV in Africa is the increase in the burden of noncommunicable diseases that was previously hidden by the high HIV-related deaths [112]. Countries in SSA already experience an increasing burden of noncommunicable diseases [113]. This will in the long run overwhelm the health care services in SSA. The risks of noncommunicable diseases have been documented to be high in SSA [114], while the prevention efforts are limited. The lifestyle has been largely westernized in SSA; the meals contain high levels of calories and smoking habit is high and still increasing.

The rates of chronic comorbid conditions have been found to be high in elderly HIV-infected patients in SSA [115]. Mortality is higher in elderly than younger HIV-infected patients [12]; immune recovery has been found to be poor [38, 116]. In addition, malnutrition has been found to be more common in elderly than younger individuals in SSA [117]. In most SSA countries, the elderly are not a priority in health care services. The priority has always been given to children under five, pregnant women, and young men living with HIV. More studies are required in SSA to better understand the challenges in this population and their needs. Studies evaluating HIV and aging in sub-Saharan Africa are summarized in Table 2.

## 7. Areas for Future Research

As a response to the change in epidemiology of HIV infection and the increase in the number of elderly HIV-infected individuals, office of AIDS Research of the National Institutes of Health (NIH) assembled a working group with the goal of assessing what is known and unknown, and what the priorities should be for research at the interface of HIV, aging, and multimorbidity. The team met face to face in 2011 and came up with several themes and areas of critical need for research. Key identified themes by the group included multimorbidity, polypharmacy, and the need to emphasize maintenance of function; the complexity of assessing HIV versus treatment effects versus aging versus concurrent disease; the interrelated mechanisms of immune senescence, inflammation, and hypercoagulability; the utility of multivariable indices for predicting outcomes; a need to emphasize human studies to account for complexity; and a required focus on issues of community support, caregivers, and systems infrastructure [119]. Future research in this population should therefore be focused on these themes, trying to fill research gaps that exist.

## 8. Conclusions and Recommendations

The number of elderly people living with HIV is on the rise globally. This increase is attributed to the effectiveness of ART that increase longevity of life but also to the increased risk of acquiring HIV in this population. Living with HIV at an advanced age is associated with a number of challenges in the range of physical, clinical, and immunological. Late presentation is common; treatment outcomes are relatively poor. Comorbidities are more common as a result of aging and HIV status. Physiological changes associated with aging alter drug metabolism in these patients who in most cases experience polypharmacy exposing them to drug toxicities and drug to drug interactions. Special designed preventive measures and screening programmes should be designed to target elderly individuals. Clinicians should also suspect HIV early in these patients to minimize late presentation. Clinicians should also pay attention to the principles of geriatric prescribing in order to minimize complications from multiple medication use. More research is needed in this population especially in sub-Saharan Africa to better understand the special needs of this population of HIV-infected individuals.

## Figures and Tables

**Table 1:** Studies evaluating immunological and clinical responses to HAART.

Study, year	Aspect studied	Main findings
Semeere et al., 2014 [44]	Immunological recovery by age	Adults younger than 50 years had on average a higher CD4 increase of 45 cells per cubic millimeter (95% confidence interval: 17 to 72; *P* = 0.001) compared with counterparts aged 60 years and older; mortality was highest among older adults compared with younger counterparts

Grabar et al., 2004 [8]	Immunologic and clinical responses to HAART in patients over 50 years old	CD4 mean increase during the first 6 months on HAART was +42.9 × 10^6^ cells/L per month in patients under 50 years and +36.9 × 10^6^ cells/L per month in patients over 50 years (*P* < 0.0001) Older patients had a significantly higher risk of clinical progression (hazard ratio (HR) = 1.52 [95% confidence interval (CI), 1.15–2.00])

Mutevedzi et al., 2011 [45]	To assess whether treatment outcomes vary with age for adults receiving antiretroviral therapy	Older adults had 32% excess mortality (*P *= * *0.004) CD4 count reconstitution was lower, despite better virological response in the older adults

Maskew et al., 2012 [46]	Differences in treatment outcomes by age category	The adjusted hazard ratios (HRs) for all-cause mortality increased with increasing age Older patients were less likely to increase their CD4 count by ≥50 cells/mm

**Table 2:** Studies evaluating aging with HIV in sub-Saharan Africa.

Study, year	Aspect studied	Main findings
Negin and Cumming, 2010 [11]	Prevalence of HIV among elderly patients	Of the approximately 21 million people in sub-Saharan Africa aged ≥15 years that were HIV+, 14.3% were ≥50 years old

Negin et al., 2012 [115]	Prevalence of HIV and chronic comorbidities among those aged 50 years and older	Rates of chronic disease were higher among all older adults compared with those aged 18–49; of those aged 50 years and older, 29.6% had two or more of the seven chronic conditions compared with 8.8% of those aged 18–49 years

Greig et al., 2012 [118]	Associations between older age and adverse outcomes in HIV/AIDS antiretroviral programmes across 17 programmes in sub-Saharan Africa	Median gain in CD4 cell count at 6 and 12 months was significantly higher in patients less than 50 years old compared with those who are at least 50 years (134 versus 112 cells/*μ*L at 6 months; 170 versus 139 cells/*μ*L at 12 months; both *P* < 0.001); in multivariate analysis, there was a significant increased risk of mortality beyond 3 months after ART initiation in all age groups of at least 40 years of age compared with less than 40 years
